# Sexual Dysfunctions in Women with Posttraumatic Stress Disorder Following Childhood Sexual Abuse: Prevalence Rates According to DSM-5 and Clinical Correlates

**DOI:** 10.1007/s10508-023-02652-0

**Published:** 2023-07-19

**Authors:** Judith Weiss, Regina Steil, Kathlen Priebe, Petra Lindauer, Nikolaus Kleindienst, Thomas Fydrich, Meike Müller-Engelmann

**Affiliations:** 1 Department of Clinical Psychology and Psychotherapy, Institute of Psychology, Goethe University Frankfurt am Main, Varrentrappstr. 40-42, 60486 Frankfurt Main, Germany; 2 Faculty of Life Sciences, Department of Psychology, Humboldt-University of Berlin, Berlin, Germany; 3 Department of Psychiatry and Psychotherapy, Charité–Universitätsmedizin Berlin, Berlin, Germany; 4 Psychology School, Hochschule Fresenius, Cologne, Germany; 5 Institute of Psychiatric and Psychosomatic Psychotherapy, Central Institute of Mental Health Mannheim, Medical Faculty Mannheim, Heidelberg University, Heidelberg, Germany; 6 Faculty of Human Sciences, Department of Psychology, Medical School Hamburg, Hamburg, Germany

**Keywords:** Female sexual dysfunctions, Posttraumatic stress disorder, Childhood abuse, PTSD cluster, DSM-5

## Abstract

Many women with posttraumatic stress disorder (PTSD) after child sexual abuse (CSA) suffer from sexual problems. However, little is known about the frequency of female sexual dysfunctions (FSD) as defined by DSM-5 among women with PTSD due to CSA. Furthermore, factors related to FSD in this patient population are understudied. To assess prevalence rates and clinical correlates of FSD according to DSM-5 criteria in women with PTSD after CSA, a structured clinical interview for sexual dysfunctions according to DSM-5 criteria was administered in a sample of 137 women with PTSD after CSA. Participants also completed measures for PTSD, depression symptoms, and borderline personality disorder symptoms. The association between FSD, severity of abuse, PTSD-, depression-, borderline symptom severity, and age was examined. In a second step, the association between FSD and PTSD-clusters was assessed. Diagnostic criteria of female sexual interest/arousal disorder (FSIAD) were met by 2.6% of women in our sample. 5.2% met criteria of female orgasmic disorder (FOD), and 11.8% those of genito-pelvic pain/penetration disorder (GPPPD). PTSD symptom severity predicted number of fulfilled criteria of FSIAD and FOD, the cluster “negative alterations in cognition and mood,” was associated with more fulfilled criteria in FSIAD and FOD. The majority of women reported sexual problems, but diagnostic criteria of FSD were met by only a small number of participants. PTSD symptoms, especially the cluster “negative alterations in cognition and mood,” seem to be related to female sexual functioning after CSA.

## Introduction

Increased rates of sexual problems have been found in individuals with a diagnosis of posttraumatic stress disorder (PTSD; Cosgrove et al., 2002; Letourneau et al., 1996) with women being more affected than men (Tagay et al., 2004). Problems with sexual functioning are associated with decreased happiness and quality of life (Laumann et al., 1999) and can hence be an additional source of suffering for patients with PTSD. Yet, female sexual problems and dysfunctions are often a neglected topic, both in therapy (Reinecke et al., 2006) and in research concerning PTSD (O'Driscoll & Flanagan, 2016).

The fifth edition of the *Diagnostic and Statistical Manual of Mental Disorders* (DSM-5) defines sexual dysfunction as “clinically significant disturbance in a person’s ability to respond sexually or to experience sexual pleasure” (American Psychiatric Association, APA, 2013). Female sexual dysfunctions (FSD) are distinguished by the areas of interest/arousal, orgasm, and pain. Each FSD’s diagnostic criteria according to DMS-5 is presented in Table 1. Notably, a duration of minimum 6 months (B-criterion), as well as clinically significant distress caused by the sexual symptoms (C-criterion) are prerequisites for the diagnosis of FSD.

**Table 1:** Diagnostic criteria for female sexual dysfunctions according to DSM-5 (APA, 2013)

	FSIAD	FOD	GPPPD
A-criterion	Lack of, or significantly reduced, sexual interest/arousal, as manifested by at least three of the following: Absent/reduced interest in sexual activity Absent/reduced sexual/erotic thoughts or fantasies No/reduced initiation of sexual activity Absent/reduced sexual excitement/pleasure during sexual activity Absent/reduced sexual interest/arousal in response to any internal or external sexual/erotic cues Absent/reduced or no genital sensations during sexual activity	Presence of either of the following symptoms: Marked delay in, marked infrequency of, or absence of orgasm Marked reduced intensity of orgasmic sensations	Persistent or recurrent difficulties with one or more of the following: Vaginal penetration or intercourse Marked vulvovaginal or pelvic pain during vaginal intercourse or penetration attempts Marked fear or anxiety about vulvovaginal or pelvic pain in anticipation of, during, or as a result of vaginal penetration Marked tension or tightening of the pelvic floor muscles during attempted vaginal penetration
B-criterion	The symptoms in criterion A have persisted for a minimum duration of approximately 6 months		
C-criterion	The symptoms in A cause clinically significant distress in the individual		
D-criterion	The sexual dysfunction is not better explained by a nonsexual mental disorder or as a consequence of severe relationship distress or other significant stressors and is not attributable to the effects of a substance/medication or another medical condition		

*FSIAD* female sexual interest arousal disorder, *FOD* female orgasmic disorder, *GPPPD*, genito-pelvic pain/penetration disorder

FSD are reported to be a very common problem for women. Prevalence rates of FSD in the general female population have been estimated as high as 40–50% (Lewis et al., 2010; McCabe et al., 2016). Yet these numbers originate from studies that did not apply DSM-5 criteria for FSD. Prevalence rates of FSD considering DSM-5 criteria are scarce. One study that applied proxy measures of DSM-5 criteria for FSD reported much lower prevalence rates of 3.6% for one or more FSD (Mitchell et al., 2016).

The way in which sexual dysfunctions are assessed is known to have implications on prevalence estimates. Hayes et al. (2008b) compared different diagnostic instruments to assess FSD and found that different prevalence rates were produced, depending especially on the inclusion of the distress criterion (C-criterion in DSM-5) and the definition of the time criterion (B-criterion in DSM-5). A change of the time criterion from “previous month” to “one month or more in the previous year” doubled prevalence rates of FSD. When sexual distress was included as a requirement in the assessment, prevalence estimates of the investigated disorders were reduced by two-thirds (Hayes et al., 2008). Similar results were found in larger prevalence studies. Prevalence rates for women’s sexual problems of 43–45% have been reported in the general population, but these rates dropped to 12–17% when sexual problems including distress were assessed (Briken et al., 2020; Shifren et al., 2008).

These methodological topics also apply to the assessment of FSD in patients with PTSD. A high occurrence of sexual dysfunction in male and female patients with PTSD has been reported (Yehuda et al., 2015). Nevertheless, only a very limited number of PTSD treatment studies assess difficulties regarding sexuality. Studies with female patients with PTSD that did asses sexual problems did not adhere to DSM-5 criteria for their assessment (O'Driscoll & Flanagan, 2016). Therefore, concrete prevalence rates of FSD in patients with PTSD are scarce. In one study that assessed sexual problems in female veterans who were treated for PTSD, 40.8% showed elevated sexual concern scores and 12.8% had clinically elevated dysfunctional sexual behavior scores (Schnurr et al., 2009).

The paucity of studies assessing FSD according to DSM-5 criteria in female patients with PTSD makes it unclear whether there are comorbidities between the two disorders. Furthermore, it is not possible to determine which psychopathological or psychosocial factors are associated with a higher risk of comorbid presentation of PTSD and FSD.

One factor that is both associated with PTSD and has also been identified as a risk factor of FSD is the experience of sexual abuse, especially child sexual abuse (CSA). A large proportion of CSA survivors develop PTSD (45–55%, according to Elklit & Christiansen, 2010). Also, FSD prevalence rates of up to 80% in women who experienced CSA have been reported (Leonard & Follette, 2002). In a review on the impact of CSA on women’s sexual health, Pulverman et al. (2018) reported prevalence rates between 25 and 59% of sexual problems in non-clinical female CSA survivors samples, and between 26 and 94% in clinical samples (Pulverman et al., 2018). Again, studies included in both reviews used different assessment methods for FSD and mostly did not adhere to DSM-5 criteria. Nevertheless, they show that sexual problems seem to be common in women who experienced CSA.

Some findings suggest that the risk of sexual problems is higher in women who experienced more severe types of CSA, involving, for example, the use of threat or force during the abuse, penetration, or a higher frequency of abuse (Briere & Elliott, 2003; Sarwer & Durlak, 1996). Furthermore, many people who experienced sexual abuse also experienced other forms of abuse (Briere & Elliott, 2003; Rellini & Meston, 2007), such as emotional or physical abuse. An additive effect of different types of abuse on sexual functioning has been suggested (Seehuus et al., 2015). In a recent literature review on the association between CSA and sexual difficulties, authors stress that CSA is related to sexual problems in clinical samples but not necessarily in non-clinical samples. Therefore, they conclude that rather than the experience of CSA, co-occurring clinical symptomatology like PTSD may contribute to the development of sexual problems (Bigras et al., 2021).

It has been shown that PTSD symptom severity is associated with sexual problems. For example, Wells et al. (2019) reported, that women with higher baseline PTSD symptomatology reported less sexual satisfaction (Wells et al., 2019). Furthermore, differential effects of PTSD clusters on sexual functioning have been suggested (O'Driscoll & Flanagan, 2016). In the DSM-5, four symptom clusters of PTSD are defined: (1) reexperiencing, (2) avoidance, (3) negative alterations in mood or cognitions and (4) symptoms of hyperarousal (APA, 2013). Findings of Schnurr et al. (2009) indicate that especially hyperarousal and numbing (which is part of negative alterations in mood) are related to sexual dysfunction. In another study on female veterans, anhedonia (part of cluster negative alterations in mood) and dysphoric arousal (part of cluster symptoms of hyperarousal) fully mediated the relationship between military sexual trauma and sexual satisfaction and function (Blais et al., 2018).

Often, the interpretation of the relationship of PTSD and sexual dysfunctions is complicated by confounding factors (Yehuda et al., 2015). PTSD shows high comorbidity rates with other mental disorders, like major depressive disorder (Gallagher & Brown, 2015) and borderline personality disorder (BPD; Scheiderer et al., 2015), especially after the experience of CSA. Elevated levels of sexual problems have been linked to both depressive disorders (Basson & Gilks, 2018; Kennedy & Rizvi, 2009) and BPD (Schulte-Herbrüggen et al., 2009; Signerski-Krieger et al., 2015; Zanarini et al., 2003). More specifically, emotion regulation problems, which are part of BPD, have been associated with reduced sexual satisfaction (Rellini et al., 2012). The influence of these disorders or corresponding symptoms on the occurrence of sexual dysfunctions in female patients with PTSD after CSA is often not accounted for in studies looking at FSD and PTSD.

Another important factor that should be controlled for is the age of the affected woman. Physiological changes in the course of life (e.g., during menopause) have an influence on female sexuality and can thus also influence or promote the occurrence of FSD (Aslan & Fynes, 2008; Thomas et al., 2018).

Taken together, existing literature shows an elevated risk for the presence of FSD in women with PTSD after CSA that is possibly influenced by different co-occurring factors such as PTSD symptom severity, depression, symptoms of BPD, or severity of the abuse.

To our knowledge, no study has assessed FSD according to DSM-5 criteria in patients with PTSD after CSA. The assessment of FSD in patients with PTSD and/or after CSA in the existing literature has been carried out with diagnostic measurements that applied different criteria especially regarding the consideration of sexual distress and the time-criterion (O'Driscoll & Flanagan, 2016; Pulverman et al., 2018). Therefore, prevalence estimates, as well as found associations with risk factors such as PTSD symptom severity or the severity of the sexual abuse in previous studies, might have been influenced by the way in which FSD were assessed. For a methodologically sound and comprehensive assessment of FSD, Meston and Bradford (2007) recommend the use of detailed clinical interviews. Gathering information on women’s psychosocial and medical history, as well as their sexual and relationship history should be included in the interview in order to gain a full picture of the presented sexual problem (Meston & Bradford, 2007).

Therefore, the first aim of the present study was to assess FSD according to DSM-5 criteria via clinical interview in female participants of a treatment study who suffered from PTSD after CSA. Following findings of Hayes (2008), Shifren (2008), and Briken (2020) that show a reduction of prevalence rates when time and/or distress criteria of DSM-5 are taken into account, we hypothesized that prevalence rates of FSD according to DSM-5 criteria in female patients with PTSD after CSA would be lower than previously reported prevalence rates of sexual problems after CSA.

Our second aim was to investigate the clinical correlates of sexual problems (severity of abuse, PTSD symptoms, depression symptoms and symptoms of BPD) in our sample, while controlling for age. Following the findings of Yehuda (2015), which show the strong influence of PTSD symptomatology on FSD, we hypothesized that PTSD symptoms would be a stronger predictor of FSD than the severity of the abuse or other psychopathological symptoms like depression or BPD symptoms.

Last, following findings that certain PTSD clusters seem to be particularly associated with sexual problems, our third aim was to assess if and how the four different PTSD clusters are associated with FSD. The PTSD clusters hyperarousal and alterations in cognition and mood have been shown to be associated with sexual functioning (Blais et al., 2018; Schnurr et al., 2009), we therefore hypothesized that these two clusters would be associated to FSD.

## Method

### Participants and Procedure

Participants originate from the treatment study “Treating Psychosocial and Neural Consequences of Childhood Interpersonal Violence in Adults” (RELEASE, German Clinical Trials Registration: DRKS00006095). The RELEASE study is a multicentre study funded by the German Federal Ministry for Education and Research. In this randomized controlled trial (RCT), the efficacy of Dialectical Behavior Therapy for PTSD (DBT-PTSD) was compared to cognitive processing therapy (CPT) in a sample of women with PTSD after childhood abuse with comorbid emotion regulation difficulties (Bohus et al., 2019).

Inclusion criteria for the RELEASE study were female sex and gender identity, a primary diagnosis of DSM-5 defined PTSD after interpersonal (sexual or physical) violence before the age of 18 years, fulfilling three or more DSM-5 criteria of BPD (including Criterion 6, i.e., affective instability), and availability for one year of outpatient therapy. Exclusion criteria were a lifetime diagnosis of schizophrenia, bipolar I disorder, intellectual disability, severe psychopathology requiring immediate treatment in a different setting (e.g., body mass index < 16.5), current substance dependence, life-threatening suicide attempts within the last 2 months, medical conditions contradicting exposure protocol (e.g., pregnancy), a highly unstable life situation (e.g., homelessness), and participation in either CPT or DBT-PTSD treatment during the last year. Recruitment occurred via the waiting lists of outpatient clinics in Berlin, Frankfurt, and Mannheim, Germany, as well as through advertisements.

For the original RCT, a total of 200 participants were randomized to receive either CPT (Müller-Engelmann et al., 2016; Resick et al., 2016) or DBT-PTSD (Bohus et al., 2013; Steil et al., 2018). Of the randomized participants, seven were later excluded due to a violation of inclusion criteria (no diagnosis of PTSD (*N* = 3), pregnant at time of randomization (*N* = 1), brain tumor (*N* = 1), established diagnosis of schizophrenia (*N* = 1), did not have a female gender identity (*N* = 1).

All participants took part in diagnostic sessions before therapy (baseline assessment), and after three, six, nine, 12, and 15 months. Diagnostic sessions were conducted at the respective participating universities and consisted of clinical interviews and questionnaires and were split in several appointments (see Bohus et al., 2019 for more information). Study participants received a 140 € compensation for their participation in the diagnostic sessions.

For the present study, PTSD, severity of abuse, BPD symptomatology, and depression measures were assessed at the baseline assessment. Sexual dysfunctions were assessed at the second diagnostic session (three months after the start of treatment). This procedure was chosen to prevent overburdening the patients during the intake assessments, and also because we did not expect sexual dysfunctions to change during the first phase of treatment. Clinical interviews were conducted by psychologists with a master’s degree who had received prior training. In the present study, we included women who indicated to have experienced CSA and who participated in the assessment of sexual dysfunctions in the RELEASE study.

Further information about the RELEASE study can be found in the study protocol (Bohus et al., 2019), and in the publication of the main results (Bohus et al., 2020).

### Assessments

#### Female Sexual Dysfunctions

To assess FSD, the “Structured Interview for Sexual Dysfunctions” (SISEX; Hoyer et al., 2014) was conducted. The SISEX is a standardized structured interview for assessing FSD according to DSM-5 criteria. The version used (RELEASE version; Bornefeld-Ettmann & Hoyer, 2014) includes an adapted introductory section that was modified to apply to women with a diagnosis of PTSD after interpersonal abuse in childhood (“In the following interview, you will be asked about various sexual problems that you may know about yourself. […] You will probably experience some questions as very intimate, and you may also be unfamiliar with talking about the following topics. If you feel uncomfortable, please tell me. […] Especially women who suffer from post-traumatic stress disorder and who have experienced sexual or physical violence often suffer from the consequences of the experiences in relation to their sexuality. […] Sexual difficulties are often an important part of a mental disorder and should therefore not be excluded from therapy.”).

The SISEX is structured in four sections. The first section includes questions on factors that can possibly influence sexual problems, including the psychosocial context of the patient’s life, relationship status and satisfaction, medical factors, medication, and religious beliefs. The interviewer is advised to take these factors into consideration during the course of the interview. Furthermore, it is assessed if the patient has been sexually active (with a partner or masturbation) in the past 6 months. If no sexual activity has occurred, the interview questions relate to the last period of sexual activity.

The following sections assess the DSM-5 criteria for the following female sexual dysfunctions: FSIAD, FOD, and GPPPD. Each section starts with at least one screening question (e.g., “During the last 6 months, did you have little or no interest in sex?”; “During the last six months, did you have problems getting sexually aroused?”) and only in the case of a positive answer, the interview for the respective section starts. The questions are then built on each other, if enough symptoms of Criterion A (see Table 1 for symptoms of the A-criteria for each FSD) are fulfilled, then Criterion C (symptoms causing clinically significant distress) is assessed. If criteria A and C are fulfilled, then Criterion B (presence of the symptoms for at least 6 months) and the Criterion D (sexual dysfunction not better explained by another mental disorder or substance use/medication) is determined. For the assessment of the Criterion D, current stressors, medication and medical/psychiatric diagnoses that could be related to FSD are assessed. Also, if applicable, relationship factors are again assessed (e.g., sexual pressure from the partner, adequate stimulation by the partner). The interviewer is then instructed to decide if these factors were more likely to be the reason for the sexual problems than the presence of a sexual dysfunction. Based on this assessment of all DSM-5 criteria while taking into account the psychosocial background of the patient as assessed in the first section, a decision is made if the primary diagnosis of a FSD was fulfilled.

The SISEX has not yet been psychometrically validated. In our sample, the SISEX sections for each FSD had good internal consistency with Cronbach’s alpha for FSIAD = 0.84, for FOD = 0.83, and for GPPPD = 0.82.

#### Posttraumatic Stress Disorder

For the diagnosis of PTSD, the index trauma (the current most distressing event) was assessed with the Life-Event Checklist (LEC-5; Weathers et al., 2013), a 17-item self-report questionnaire screening for traumatic events during lifetime. To be included in the present study, at least one event of CSA had to be reported. CSA was defined as the experience of any form of unwanted sexual experience before the age of 18, including kissing or touching the perpetrator, penetration (oral, vaginal, anal), or watching or performing sexual acts.

##### PTSD Symptom Severity

The severity of PTSD symptoms was assessed with the clinician administered PTSD-scale for DSM-5 (CAPS-5; Weathers et al., 2018) in which 20 symptoms are assessed on a 5-point Likert scale, ranging from 0 (*no impairment*) to 4 (*extreme impairment*). The total score of the CAPS can range from 0 to 80, with higher scores indicating greater symptom severity. The scale was used in its German version, which has been psychometrically validated (Müller-Engelmann et al., 2020).

##### PTSD Clusters

Symptom clusters of PTSD were also assessed with the CAPS-5 (Müller-Engelmann et al., 2020; Weathers et al., 2018). CAPS-5 items can be assigned to PTSD symptom clusters according to the DSM-5. The cluster Reexperiencing consists of 5 items and its score ranges from 0 to 20; the cluster Avoidance consists of 2 items (range 0–8); the cluster Negative alterations in mood or cognitions consists of 7 items (range 0–28); and the cluster symptoms of hyperarousal consists of 6 items (range 0–24). Higher scores indicate greater symptom severity in each cluster. Internal consistency of the CAPS-5 was acceptable in our sample with Cronbach’s alpha = 0.65 and McDonald’s omega = 0.69.

#### Severity of Abuse During Childhood

To assess the severity of abusive events during childhood, the Childhood Trauma Questionnaire (CTQ; Bernstein et al., 1997) was administered. This self-report questionnaire consists of 28 items assessing the frequency of maltreatment in childhood on a 5-point Likert scale (1 = “*not at all*”, 5 = “*very often*”). The items can be combined into five subscales: physical abuse, physical neglect, sexual abuse, emotional abuse, and emotional neglect, each ranging from 5 to 25. Additional three items form the minimization/denial subscale. The CTQ items can also be combined to an overall score consisting of the sum scores of the subscales, except the minimization/denial subscale. The overall score can range from 25 to 125 with higher scores indicating a higher severity of adverse events during childhood. We used this sum score of the CTQ to account for the additive effect of different types of abuse. The CTQ was administered in its German version, which has been psychometrically validated (Wingenfeld et al., 2010). Internal consistency of the CTQ in our sample was good with Cronbach’s alpha = 0.78 and McDonald’s omega = 0.79.

#### Symptoms of Depression

The German version of the Beck-Depression Inventory-II (Beck et al., 1996; Hautzinger et al., 2006) was used to assess depressive symptoms. The BDI-II consists of 21 items and its sum score can range from 0 to 92, with higher scores indicating higher depressive symptomatology. The German version of the BDI-II has been validated (Kühner et al., 2007). In our sample, internal consistency of the BDI-II was very good with Cronbach’s alpha = 0.88 and McDonald’s omega = 0.89.

#### Symptoms of Borderline Personality Disorder

To assess BPD symptoms, the German version of the Borderline Symptom List (BSL-23; Bohus et al., 2009) was used. The BSL-23 is a self-report questionnaire with 23 items answered on a 5-point Likert scale from 0 (*not at all*) to 4 (*very strongly*). The total score of the BSL-23 is calculated as the mean score and can hence range from 0 to 4. The BSL-23 is a short version of the BSL-95 which is based on the criteria for BPD of the DSM-IV. The German version of the BSL-23 has been validated and displayed good psychometric properties (Bohus et al., 2009). Internal consistency of the BSL-23 was excellent in the present sample with Cronbach’s alpha = 0.92 and McDonald’s omega = 0.93.

### Statistical Analyses

Data analyses were completed using *R* version 4.2.1. The first aim was to assess the prevalence of each FSD in our sample. For this purpose, the number of women who met the diagnoses of FSIAD, FOD and/or GPPPD, as assessed by the SISEX, was counted. Furthermore, the number of positive screening questions, as well as the number of criteria fulfilled for each disorder, was counted.

The second aim was to analyze the associations between FSD and PTSD, depression, and borderline symptom severity, severity of abuse, and age. As the number of participants fulfilling all criteria of one FSD was too small (see Results section) to assess their association to the factors of interest inference-statistically, we decided to operationalize the outcome variables as count variables. We created one count variable for each sexual dysfunction (FSIAD, FOD, GPPPD), counting the number of fulfilled criteria in the SISEX. The count variables can take a value of 0 (screening question negated), 1 (screening item endorsed, no further criteria fulfilled), 2 (screening item endorsed, A-criterion fulfilled), 3 (screening item endorsed, Criterion A and Criterion C fulfilled), 4 (screening item endorsed, Criteria A, B, and C fulfilled) or 5 (screening item endorsed, all criteria fulfilled = full diagnosis).

We calculated Pearson correlations between all predictors to check for possible multicollinearity. If Pearson correlation coefficients between predictors are close to or higher than 0.8, multicollinearity is likely to exist (Shrestha, 2020). We then used Poisson regression and negative binomial regression analyses with the count variables of each FSD as the respective dependent variable and severity of abuse, PTSD symptom severity, depression symptom severity, borderline symptom severity and age as predictor variables.

In Poisson regression, the coefficients indicate the log value change of the outcome variable when the coefficient changes by one unit. When exponentiated, one receives the incident rate ratio (IRR) for each coefficient, representing the proportional change in the outcome variable for each unit of change in the predictor (Hilbe, 2014). As IRRs are more intuitive to interpret, we report IRRs for each coefficient.

One assumption of Poisson regression is that the distribution mean and variance are equal. When this assumption is violated, i.e., the variance of the Poisson model exceeds the mean, the model shows overdispersion (Hilbe, 2014). To check for overdispersion, the model dispersion statistic can be calculated, defined as the Pearson residuals squared divided by the model residual degrees of freedom. If the dispersion statistic is greater than 1, overdispersion is indicated (Hilbe, 2014). If overdispersion is present, negative binomial regression has been shown to be an appropriate and frequently used alternative, as the variance of the negative binomial distribution is larger than that of Poisson and can thus handle overdispersion (Hilbe, 2014; Ismail & Jemain, 2007).

In our models, dispersion statistics were 0.91 in the model for FSIAD, 1.13 in the model for FOD, and 2.51 in the model for GPPPD, with overdispersion indicated in the FOD and GPPPD models. We therefore performed negative binomial regression in the models with FOD and GPPPD count variables as dependent variables.

To assess the relationship between the four PTSD clusters and FSD, we again performed Poisson regression analyses (FSIAD) and negative binomial regression analyses (FOD and GPPPD). Instead of a sum score of PTSD symptom severity, the four PTSD cluster scores were entered as explanatory variables in the models.

## Results

In the current study, 137 participants (53.3% (*N* = 73) from the DBT-PTSD group and 46.7% (*N* = 64) from the CPT group) were included, as they had experienced CSA and participated in the diagnostic session with the SISEX. From the original sample (*N* = 193), 15.5% (*N* = 30) had dropped out before the second diagnostic assessment, 10.9% (*N* = 15) indicated they had not experienced CSA, and 6.7% (*N* = 11) declined participation of the SISEX due to the following reasons: never experienced any sexuality (*N* = 1), did not want to talk about sexuality (*N* = 3), no reason given (*N* = 7).

Descriptive statistics of the 137 participants can be found in Table 2. Participants had mean PTSD symptom severity scores of 40.58 on the CAPS-5 (*SD* = 9.47), mean depression symptom severity scores of 33.96 on the BDI-II (*SD* = 10.63) and mean borderline symptom severity scores of 2.06 on the BSL-23 (*SD* = 0.75). The mean score of severity of abuse was 78.72 (*SD* = 19.82) on the CTQ.

**Table 2:** Participant characteristics (n = 137)

Characteristic	Participants, n (%)
Age, mean (SD) in years	36.12 (11.1)
Marital status	
Single	66 (48.1)
In partnership or married	34 (24.8)
Divorced or separated	34 (24.8)
Widowed	2 (1.5)
No information	1 (.7)
Children (> = 1)	65 (47.5)
Sexual orientation	
Heterosexual	97 (70.8)
Homosexual	12 (8.8)
Bisexual	28 (20.4)
Unclear	0 (0)
Sexual activity during last 6 months No sexual activity during last 6 months	93 (67.9) 44 (32.1)

### Prevalence of Female Sexual Dysfunctions According to DSM-5 Criteria

**Fig. 1:**
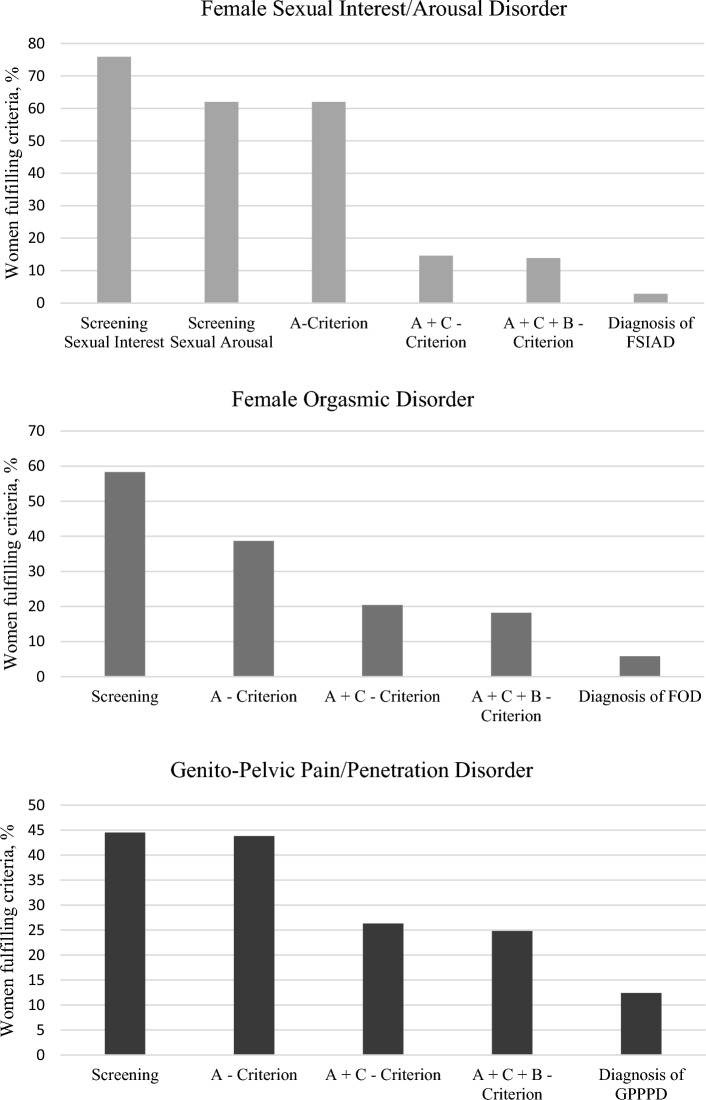
Percentage of women (N = 137) affirming the screening items and met DSM-5 criteria for FSD as diagnosed by the SISEX. *Note* A-criterion: refers to the specific criteria defined for each female sexual dysfunction in the DSM-5; C-criterion: The symptoms in A cause clinically significant distress; B-criterion: The symptoms in A have persisted for a minimum of 6 months

Figure 1 gives an overview of all confirmed diagnostic criteria for each sexual dysfunction according to DSM-5. Criteria for the diagnosis of FSIAD were met in 2.9% of the cases (*N* = 4). In 5.8% of the cases (*N* = 8) criteria for the diagnosis of FOD were met. The GPPPD diagnosis was met by 12.4% (*N* = 17). With respect to comorbidities within sexual dysfunctions, one woman met diagnostic criteria for all three disorders, one woman met criteria for both Female Sexual Interest/Arousal Disorder and Female Orgasmic Disorder, one for Female Sexual Interest/Arousal Disorder and Genito-Pelvic Pain/Penetration Disorder, and one for Female Orgasmic Disorder and Genito-Pelvic Pain/Penetration Disorder.

#### Endorsed Criteria for Each Female Sexual Dysfunction

With a closer look at the different criteria for each FSD, it became noticeable that both the screening questions and Criterion A were endorsed and met by a much greater number of women than the full diagnostic criteria.

##### Female Sexual Interest/Arousal Disorder

The screening item for reduced sexual interest was endorsed by 75.9% of women and the screening item for reduced sexual arousal by 62.0%. Criterion A was met by 62.0%, indicating they reported at least three symptoms of reduced sexual interest and/or arousal. While only 14.6% reported to suffer from these symptoms (Criterion C), most of these women also met the time criterion (Criterion B). In only 2.9% of the cases, the interviewer decided that the diagnosis was applicable, and the disorder was not better explained by other factors, such as PTSD, depression, or medication (Criterion D).

##### Female Orgasmic Disorder

A similar picture is evident for FOD: a larger number of women endorsed the screening item, indicating they experienced difficulties achieving orgasm (58.4%) and met Criterion A (38.7%). Only 20.4% reported suffering from it, most of them for at least 6 months (18.2%). Nine women did not complete the FOD section of the SISEX.

##### Genito-Pelvic Pain/Penetration Disorder

For GPPPD, 44.5% endorsed the screening item, indicating that they experienced pain during penetration and almost all those women also met the Criterion A (43.8%). Compared to FSIAD and FOD, more women indicated to suffer from GPPPD symptoms: more than half of the women met Criterion A for GPPPD also suffered from pain during penetration (26.3%), mostly at least for the past 6 months (24.8%).

### Associations Between Explanatory Variables and Number of Fulfilled Criteria of Female Sexual Dysfunction

In Table 3 Pearson correlation coefficients between the predictor variables are displayed. None of the correlation coefficients were > 0.8, indicating that multicollinearity between predictors is unlikely.

**Table 3:** Pearson regression coefficients of predictor variables

	PTSD symptom severity	BPD symptom severity	Depression symptom severity	Severity of abuse
PTSD symptom severity	–	.34	.36	.18
BPD symptom severity		–	.62	.12
Depression symptom severity			–	.11
Severity of Abuse				–

Table 4 shows the results of the three regression models, with the count variable for the respective diagnosis of FSD as outcome variable. The Poisson regression analyses revealed that in the FSIAD model, PTSD symptom severity (IRR = 1.02, 95% CI [1.00, 1.04], *p* < 0.05) and depression symptom severity (IRR = 1.02, 95% CI [1.00, 1.04], *p* < 0.05) were significantly related to the number of fulfilled criteria. Each unit increase in PTSD symptom severity (CAPS sum score) increased the number of FSIAD fulfilled criteria by a factor of 1.02. The same direction of results emerged for depression symptom severity.

**Table 4:** Results of poisson regression analyses (for FSIAD) and negative binomial regression analyses (for FOD and GPPPD)

Variable	FSIAD		FOD		GPPPD	
	Est	IRR [95% CI]	Est	IRR [95% CI]	Est	IRR [95% CI]
Age (years)	.00	1.00 [.99, 1.01]	–.02	.98 [.96, 1.00]	–.04**	.96 [.94, .99]
Severity of abuse	.00	1.00 [.99, 1.01]	–.00	1.00 [.99, 1.01]	.00	1.00 [.99, 1.02]
PTSD symptom severity	.02*	1.02 [1.00, 1.04]	.03**	1.03 [1.01, 1.06]	.02	1.03 [.99, 1.06]
Depression symptom severity	.02*	1.02 [1.00, 1.04]	.01	1.01 [.99, 1.04]	.03	1.03 [.99, 1.07]
BPD symptom severity	–.19	.83 [.66, 1.04]	–.20	.82 [.57, 1.16]	–.17	.85 [.52, 1.37]

*IRR* incident rate ratio, *FSIAD* female sexual interest/arousal disorder, *FOD* female orgasmic disorder, *GPPPD* genito-pelvic pain/penetration disorder, *CSA* child sexual abuse, *CPA* child physical abuse, *PTSD* posttraumatic stress disorder, *BPD* borderline personality disorder

* *p* < .05; ** *p* < .01; *** *p* < .001

In the negative binomial regression model for FOD, PTSD symptom severity (IRR = 1.03, 95% CI [1.01, 1.06], *p* < 0.01) was significantly associated with number of FOD symptoms: Each unit increase of PTSD symptom severity increased the number of fulfilled FOD criteria by a factor of 1.03. In the GPPPD negative binomial regression model, only age (IRR = 0.96, 95% CI [0.94, 0.99], *p* < 0.01) showed a significant association with the number of sexual symptoms. Each year increase in age decreased the number of GPPPD criteria by a factor of 0.96. Severity of abuse as well as BPD symptom severity were not associated with the number of fulfilled criteria of any of the three sexual dysfunctions.

### Associations Between Posttraumatic Stress Disorder Clusters and Number of Fulfilled Criteria of Female Sexual Dysfunction

In the model for FSIAD, negative alterations in mood and cognition emerged as the only significant predictor (IRR = 1.26, 95% CI [1.06, 1.49], *p* < 0.01), indicating that each unit increase in this PTSD-cluster increased the number of fulfilled FSIAD criteria by a factor of 1.26. Alterations in mood and cognition also significantly predicted number of fulfilled FOD criteria (IRR = 1.35, 95% CI [1.05, 1.76], *p* < 0.05). A one unit increase in the PTSD cluster negative alterations in mood and cognition increased the number of fulfilled criteria of FOD by a factor of 1.35. In the GPPPD model the association between number of fulfilled criteria and negative alterations in mood and cognition almost reached significance (IRR = 1.39, 95% CI [0.98, 2.01], *p* = 0.05). Again, age significantly predicted number of fulfilled GPPPD criteria. Table 5 summarizes the results of the three models.

**Table 5:** Results of poisson regression analyses (for FSIAD) and negative binomial regression analyses (for FOD and GPPPD) with PTSD-clusters as predictors

Variable	FSIAD		FOD		GPPPD	
	Est	IRR [95% CI]	Est	IRR [95% CI]	Est	IRR [95% CI]
Age (years)	.00	1.00 [.99, 1.01]	–.02	.98 [.96, 1.00]	–.04**	.96 [.94, .99]
Severity of abuse	.00	1.00 [.99, 1.01]	.00	.99 [.98, 1.01]	.00	1.00 [.99, 1.02]
PTSD clusters Re-experiencing Avoidance Negative alterations in mood/cognition hyperarousal	.05 .06 .23** –.07	1.05 [.91, 1.21] 1.06 [.92, 1.23] 1.26 [1.06, 1.49] .93 [.80, 1.09]	.10 –.06 .30* .07	1.11 [.89, 1.38] .94 [.75, 1.18] 1.35 [1.05, 1.76] 1.07 [.85, 1.36]	–.19 .03 .33 .19	.82 [.62, 1.09] 1.03 [.76, 1.39] 1.39 [.98, 2.01] 1.22 [.88, 1.69]
Depression symptom severity	.14	1.15 [.96, 1.39]	.07	1.07 [.80, 1.42]	.27	1.32 [.89, 1.95]
BPD symptom severity	–.11	.90 [.75, 1.07]	–.16	.85 [.65, 1.11]	–.13	.88 [.61, 1.25]

*IRR* incident rate ratio, *FSIAD* female sexual interest/arousal disorder, *FOD* female orgasmic disorder, *GPPPD* genito-pelvic pain/penetration disorder, *CSA* child sexual abuse, *CPA* child physical abuse, *PTSD* posttraumatic stress disorder, *BPD* borderline personality disorder

* *p* < .05; ** *p* < .01; *** *p* < .001

## Discussion

The aim of this study was to assess the prevalence of FSD according to DSM-5 criteria in a sample of women with PTSD after CSA and to assess clinical correlates of FSD in this population. In our sample of women between 2.9 and 12.4% met criteria for at least one of the three sexual dysfunction diagnoses according to DSM-5. We thus found much smaller prevalence rates of full FSD diagnoses than previously reported in studies that did not consider distress or time criteria (Leonard & Follette, 2002; Pulverman et al., 2018). Furthermore, in our sample, prevalence was lowest for FSIAD; only 2.9% of women met diagnostic criteria for this disorder. Compared to previous findings that report dysfunctions of sexual interest and/or arousal to be the most common FSD in general (McCabe et al., 2016) as well as in women who experienced CSA (Pulverman et al., 2018), this finding is surprising. The most frequently fulfilled diagnosis in our sample was GPPPD, which was diagnosed in 12.4% of women. This result is in line with the literature reporting sexual pain to be the most prevalent sexual problem in female veterans (Pulverman & Creech, 2021), even though not all women in the included studies had experienced sexual abuse.

A reason for the lower prevalence rates found in our study is most likely the way in which we assessed and diagnosed FSD. We conducted a detailed clinical interview, in which all DSM-5 criteria were taken into consideration, including the required duration of symptoms and related distress, as well as possible influential factors (e.g., partnership factors, medication, psychiatric disorders). In prior studies, such an exact diagnostic process was most often not pursued. Instead, sexual functioning has often been assessed via subscales from measures of wider trauma symptoms or self-report instruments (e.g., in studies included in the review by O’Driscoll & Flanagan (2016)). This way of assessing sexual functioning seems to capture sexual problems rather than a diagnosis of FSD (according to DSM-5) and could be the major reason for differences between our numbers and previously reported prevalence rates. This is also reflected in our results pattern: the majority of women actually reported sexual problems as indicated by the high number of endorsed screening questions for all FSDs. 82.5% of participants endorsed screening questions for FSIAD, 58.4% for FOD, and 44.5% for GPPPD. These screening questions equal short, one item self-report assessments that have been used to assess FSD in previous studies and hence correspond more closely to the higher prevalence rates reported for example in the literature review by Pulverman et al. (2018). The rates in clinical samples of women with CSA experiences range between 18 and 62% for desire and arousal problems, between 9 and 37% for sexual pain and between 18 and 45% for orgasm problems. In our study, when FSD assessment became more fine-grained and followed DSM-5 criteria, fewer women met all criteria. Especially when the distress criterion was assessed, the number of women who endorsed the criterion dropped. For example, out of 85 women who met FSIAD Criterion A, only 20 reported also experiencing distress. This goes in line with previous reports (Briken et al., 2020; Hayes et al., 2008; Shifren et al., 2008) that the assessment of the distress criterion reduces prevalence rates up to two-thirds. For GPPPD, this difference was not as pronounced. Most women who reported sexual pain also experienced distress from it and were more likely to meet the full diagnostic criteria for GPPPD. This could be one reason why GPPPD had the highest prevalence rate in our sample.

Explanations why women in our sample did not experience distress from reported sexual problems could possibly be due to an avoidance of sexual contacts. It has been reported that women with PTSD show high levels of sexual avoidance (e.g., Büttner et al., 2014). Especially women who experienced sexual abuse have been found to avoid sexual relationships and sexual contacts (van Berlo & Ensink, 2000). If a woman with, for example, low desire avoids sexual relationships and therefore does not feel sexual distress, diagnostic criteria of FSIAD according to DSM-5 are not met. In our sample, 68% of women indicated that they had been sexually active in the past 6 months (including both masturbation and/or sexual activity with a partner). In the 32% of women who indicated that they had not been sexually active, it seems plausible that avoidance of sexuality played a decisive role. At this point, the DSM-5 criteria are not ideal to cover the complexity of female sexual functioning after trauma as sexuality avoidance is not thematized in the DSM-5 criteria for FSD.

Another reason why women in our sample seldom indicated that they experienced distress from sexual problems could be that our sample consisted of women highly affected by PTSD, and symptoms of depression and BPD. Hypothetically, problems with sexuality seem secondary to the affected women to more pressing problems connected to this symptomatology. If this was the case, one could expect that problems with sexuality will become more relevant towards the end of a treatment, when PTSD and depression hopefully remit. This consideration could be examined in future studies.

Regarding clinical correlates of sexual dysfunction in women with PTSD after CSA, we found PTSD symptomatology to predict the number of FSIAD and FOD symptoms. Therefore, women with more severe PTSD symptoms were likely to meet more diagnostic criteria of one of these sexual disorders. In addition to PTSD symptoms, higher depression predicted a higher number of fulfilled FSIAD criteria. This underscores the relevance of depression in the context of female sexual arousal and desire dysfunctions in our sample, and is consistent with other studies where depressive symptoms have been found to be an important factor in the prediction of low sexual desire in women who experienced childhood trauma (O'Loughlin et al., 2020). Severity of depression symptoms was not related to the number of fulfilled FOD and GPPPD criteria. Age was a relevant predictor for the number of fulfilled GPPPD diagnostic criteria. Interestingly, older age was associated with less GPPPD symptoms. This is contrary to previous findings that find increased sexual dysfunction in older age (Dennerstein & Hayes, 2005). One explanation for this could be a decline in sexual activity with older age (Dennerstein et al., 2003; Hess et al., 2009). When sexual activity declines, prior symptoms of sexual pain may not play an important role in women’s life anymore, and hence they do not suffer that much from them.

To further analyze the relationship between PTSD and FSD, we examined PTSD symptom clusters and their association to FSD and found that only the cluster negative alterations in mood and cognition emerged as a predictor of FSIAD and FOD criteria. These results are in line with other findings on the relevance of this particular PTSD symptom cluster for sexual dysfunction. Blais et al. (2018) found that negative alterations in cognition and mood explained the relationship between military sexual trauma and sexual function and satisfaction in veteran women. Additionally, this cluster also predicted higher rates of sexual dysfunction in male veterans (Letica-Crepulja et al., 2019). Schnurr et al. (2009) found that numbing (part of negative alterations in mood and cognition) was related to dysfunctional sexual behavior and sexual concerns. Possible explanations for the relationship between this PTSD cluster and FSD could be related to the content of the altered cognitions after the experience of a trauma. After sexual abuse, negative alterations in cognition might include negative beliefs about sexuality in general (“sex is disgusting”) or about one’s own sexual self-concept. The association between a negative sexual self-concept and sexual dysfunction has been shown before (Meston et al., 2006). Also, after non-sexual trauma, negative cognition alterations might affect sexuality, including dysfunctional thoughts like “I am to blame for what happened, therefore I do not deserve to enjoy sexuality” or negative beliefs about others (“People always want to take advantage of me”) could interfere with sexual functioning (Bornefeld-Ettmann & Steil, 2017).

### Clinical Implications

Translated into clinical practice, a thorough anamnesis of sexuality should be part of a treatment of female patients with PTSD, especially with PTSD after CSA. Even though prevalence rates for FSD in women with PTSD after CSA were lower in our study than those of sexual problems in previous research, this does not mean topics related to sexuality or sexual functioning can be neglected in therapy. Our data rather shows how complex the association of sexual problems and PTSD, CSA, and comorbid clinical symptoms is. To account for this complexity, therapists should take time to carefully inquire about potential sexual challenges. Many women with PTSD after CSA might indicate a loss of desire or difficulties achieving orgasm (such as the positive screening questions in our sample). The implication of these topics on each woman’s individual life should be inquired in therapy. In our study, many women reported not suffering from e.g., a loss of sexual desire. Then, of course, these issues should not be pathologized. Rather, a space should be provided to talk about possible concerns if they are present. Topics related to sexuality, such as a wish for intimacy, the pressure to be sexually active (e.g., by society or a partner), the meaning of sexuality for the woman’s personal life, or negative cognitions about one’s own sexuality might be important topics to address in this context.

Our findings also indicate that cognitions that have been altered by traumatic experiences seem to influence women’s sexuality. Hence, therapeutic strategies that target dysfunctional cognitions and other symptoms of this PTSD cluster will potentially have a beneficial effect on sexual functioning in women with PTSD. More research on this connection and possible further therapeutic interventions is needed.

### Limitations

There are several limitations to our study. First, our sample was rather small for a prevalence study. Also, it consisted of a specific population of women with PTSD and emotion regulation difficulties after CSA. For the purpose of the parent trial (the RELEASE study), participants had to fulfill three DSM-5 criteria of BPD including the criterion “affective instability.” This special feature of our sample may limit the generalizability of our results.

Second, the assessment of FSD took place three months after therapy had already started. As data from this study stem from a large RCT that was designed to compare the efficacy of DBT-PTSD against CPT, participants had to complete a multitude of interviews and questionnaires at the baseline assessment. The baseline diagnostic session was therefore already long, and participants were at this point still unfamiliar with the diagnostic process and the diagnostic staff. To avoid overburdening the participants we decided to assess FSD (including the assessment of intimate details about their sexual life) at the second diagnostic session, three months later. It is possible that the symptomatology of FSD could have changed in the period between the baseline assessment and this second diagnostic session. In this time-period, participants received up to 12 h of therapy. Based on the content of these first 12 sessions in both treatment groups, we assume that it is unlikely that sexual dysfunction changed during this time. In both treatment groups the first four sessions were reserved for biographical history taking and to work out an emergency plan. In the CPT group, the focus of the first eight sessions was psychoeducation, determination of stuck points, and the examination and modification of problematic thoughts and beliefs. Whereas it cannot be ruled out that these topics influenced FSD symptomatology, topics that are more closely related to sexuality such as "being close to other people" or "trust", were addressed in later sessions. In DBT-PTSD, the first sessions are part of the treatment phases “commitment” and “trauma model and motivation” (Bohus et al., 2019). These include an introduction to the skills concept as well as to mindfulness, psychoeducation about PTSD, and analysis of avoidance and escape strategy. Mindfulness-based interventions have been shown to have a positive effect on women’s sexual functioning (Brotto & Smith, 2014). Hence, it is possible that in the DBT-PTSD group, mindfulness interventions had a positive effect on sexual functioning that was not present in the CPT group.

Third, the psychometric properties of the clinical interview used in our study, the SISEX, have not yet been examined. This poses questions about the validity and reliability of this instrument. Related to this, the application of the Criterion D when diagnosing FSD with the SISEX in a sample of women who all suffer from PTSD should be critically questioned. Criterion D requires that the FSD cannot be better explained by another psychiatric disorder, but there are no clear guidelines how to evaluate this. In the end, it is the individual decision of the clinician who runs the SISEX if the FSD is better explained by PTSD or comorbid disorders. Nevertheless, the use of the SISEX allowed for a very detailed examination of sexual functioning and related factors in our sample. Furthermore, other validated clinical interviews for FSD are scarce and hence the SISEX was the best available alternative for us. Future studies should make an effort to validate existing diagnostic measures like the SISEX.

### Conclusions

The present study is to our knowledge the first to assess FSD according to DSM-5 criteria in a sample of women with PTSD after CSA. Even though sexual problems were indicated by the majority of participants, full FSD diagnoses were met by a small number of women. Taken together, the most important implication of our study is that sexuality should always be discussed and be part of therapy in female patients with PTSD. It is not enough to ask simple yes/no questions, as they do not do justice to the complexity of the topic. A more in-depth exploration is required, in which the individual circumstances (e.g., partnership, medical factors, psychosocial factors) are considered, and in which the sexual problem with its consequences and possible resulting distress are elicited. This is important to really understand sexual problems and dysfunctions and to take them into account adequately in the treatment. Also, more research on the topic is necessary, to further understand the interplay between PTSD, CSA, comorbid disorders, and female sexual functioning.

## Data Availability

The data that support the findings of this study is available from the corresponding author upon reasonable request. The data is not yet publicly available as it is part of the RELEASE study (Bohus et al., 2019) and within this study will be used for further publications.
